# Mac-2 Binding Protein Is a Novel E-Selectin Ligand Expressed by Breast Cancer Cells

**DOI:** 10.1371/journal.pone.0044529

**Published:** 2012-09-06

**Authors:** Venktesh S. Shirure, Nathan M. Reynolds, Monica M. Burdick

**Affiliations:** 1 Department of Chemical and Biomolecular Engineering, Russ College of Engineering and Technology, Ohio University, Athens, Ohio, United States of America; 2 Biomedical Engineering Program, Russ College of Engineering and Technology, Ohio University, Athens, Ohio, United States of America; University of Kentucky College of Medicine, United States of America

## Abstract

Hematogenous metastasis involves the adhesion of circulating tumor cells to vascular endothelium of the secondary site. We hypothesized that breast cancer cell adhesion is mediated by interaction of endothelial E-selectin with its glycoprotein counter-receptor(s) expressed on breast cancer cells. At a hematogenous wall shear rate, ZR-75-1 breast cancer cells specifically adhered to E-selectin expressing human umbilical vein endothelial cells when tested in parallel plate flow chamber adhesion assays. Consistent with their E-selectin ligand activity, ZR-75-1 cells expressed flow cytometrically detectable epitopes of HECA-452 mAb, which recognizes high efficiency E-selectin ligands typified by sialofucosylated moieties. Multiple E-selectin reactive proteins expressed by ZR-75-1 cells were revealed by immunoprecipitation with E-selectin chimera (E-Ig chimera) followed by Western blotting. Mass spectrometry analysis of the 72 kDa protein, which exhibited the most prominent E-selectin ligand activity, corresponded to Mac-2 binding protein (Mac-2BP), a heretofore unidentified E-selectin ligand. Immunoprecipitated Mac-2BP expressed sialofucosylated epitopes and possessed E-selectin ligand activity when tested by Western blot analysis using HECA-452 mAb and E-Ig chimera, respectively, demonstrating that Mac-2BP is a novel high efficiency E-selectin ligand. Furthermore, silencing the expression of Mac-2BP from ZR-75-1 cells by shRNA markedly reduced their adhesion to E-selectin expressing cells under physiological flow conditions, confirming the functional E-selectin ligand activity of Mac-2BP on intact cells. In addition to ZR-75-1 cells, several other E-selectin ligand positive breast cancer cell lines expressed Mac-2BP as detected by Western blot and flow cytometry, suggesting that Mac-2BP may be an E-selectin ligand in a variety of breast cancer types. Further, invasive breast carcinoma tissue showed co-localized expression of Mac-2BP and HECA-452 antigens by fluorescence microscopy, underscoring the possible role of Mac-2BP as an E-selectin ligand. In summary, breast cancer cells express Mac-2BP as a novel E-selectin ligand, potentially revealing a new prognostic and therapeutic target for breast cancer.

## Introduction

The five-year survival rate for breast cancer patients is almost 98% if the disease is detected in early stages. However, if the primary growth has metastasized to distant organs, the survival rate decreases drastically to 27% [1]. This bleak statistic emphasizes a need for greater understanding and better interventions for the prevention of metastasis. Metastatic invasion to distant organs is a systematic series of events, in which cancer cells dissociate from a primary tumor, enter the circulatory system, travel through the vasculature, attach to endothelium of a specific secondary site, and traverse the vascular wall to colonize the tissue. It is believed that the attachment of circulating tumor cells to endothelium occurs through a mechanism that is similar to the recruitment of leukocytes to inflamed tissue. According to this model, flowing leukocytes form initial contacts (capture), which lead to continuous but transient interactions (rolling), and finally arrest of the cells on endothelium (firm adhesion). E-selectin expressed by endothelial cells is a well-recognized mediator of adhesion of cancer cells and cells of hematopoietic origin [2], [3], [4], [5], [6], [7], [8], [9], [10]. E-selectin engages its counter-receptors expressed on flowing cells, which not only captures and slows down the cells but also activates other mechanisms that promote tissue homing [2], [11], [12], [13]. The significance of E-selectin mediated interactions in metastasis is apparent in several *in vivo* studies, wherein metastasis in mice was reduced when E-selectin and/or E-selectin ligand activity were blocked, compared to control conditions [14], [15]. Therefore, understanding of E-selectin ligands expressed on cancer cells may be critical in devising new prognostic and therapeutic strategies against cancer metastasis.

Several E-selectin ligands have been identified on human colon cancer, prostate cancer, leukemic, and hematopoietic cells, such as CD43, PSGL-1, CD44, PCLP, and CEA [2], [4], [16], [17], [18], [19], [20]. Although diverse proteins appear to function as E-selectin ligands, the core species of these ligands are primarily decorated with sialofucosylated carbohydrates such as the tetrasaccharide sialyl Lewis X (sLe^x^) and its stereoisomer sialyl Lewis A (sLe^a^), both of which are detectable by the HECA-452 monoclonal antibody (mAb) [6], [16], [21], [22]. In general, glycoproteins recognized by HECA-452 mAb are believed to be high affinity E-selectin ligands [6], [16], [21]. Breast cancer cell lines possess E-selectin ligand activity and are known to express sLe^x^, sLe^a^, and HECA-452 mAb reactive oligosaccharides [7], [8], [23], [24], [25], [26], [27]. Recently, we identified sialylated glycolipids (gangliosides) as breast cancer cell E-selectin ligands [23]. However, core glycoprotein E-selectin ligands recognized by HECA-452 mAb and expressed by breast cancer cells remain to be elucidated and are the focus of the work herein.

Because reports identifying breast cancer glycoprotein E-selectin ligands are lacking, it is anticipated that breast cancer cells may express novel ligands. Of potential interest is Mac-2BP, a highly glycosylated protein that has been recognized as a cell adhesion molecule [28], [29], [30], but which has never been tested for E-selectin ligand activity. Notably, breast cancer patients with Mac-2BP over-expressing tumors are more likely to develop distant metastasis compared to patients with low Mac-2BP expressing tumors [31]. To explain the poor prognosis in the former type of patients, the study authors postulated that Mac-2BP acts as a cell adhesion molecule that promotes tumor cell interactions with vascular endothelium [31]. In addition, Mac-2BP is a well-documented counter-receptor of galectin-1 (Gal-1) [32], and some reports suggest cross-reactivity of Gal-1 ligands with E-selectin [17], [33]. For instance, CD43, a Gal-1 ligand expressed by T-cells, is also an E-selectin ligand [17], [33]. Despite these lines of evidence, neither E-selectin activity nor any other functional or mechanistic role of Mac-2BP in promoting tumor metastasis has been reported.

In this study, we investigated the E-selectin ligands expressed by the bone metastatic ZR-75-1 breast carcinoma cell line [34]. We identified and characterized Mac-2BP as a novel high affinity E-selectin ligand on ZR-75-1 cells, and also tested its presence on several other metastatic breast cancer cell lines and in pathological tissue samples. Ultimately, knowledge of E-selectin ligands expressed by breast cancer cells may lead to novel molecular tools to inhibit metastasis.

## Materials and Methods

### Cell Culture

Except where indicated, cell lines were obtained from the American Type Culture Collection (ATCC, Manassas, VA). ZR-75-1 and T-47D breast cancer cell lines were cultured in RPMI (Life Technologies, Carlsbad, CA) supplemented with 10% fetal bovine serum (FBS) and 1x penicillin-streptomycin (Life Technologies). The BT-20 breast cancer cell line was grown in minimum essential medium (MEM; Life Technologies) with 10% FBS and 1x penicillin-streptomycin. Hs-578t, MDA-MB-231, MDA-MB-468, and MCF-7 breast cancer cells were maintained in Dulbecco’s modified eagle medium (Life Technologies) with 15% FBS and 1x penicillin-streptomycin. E-selectin transfected Chinese hamster ovary cells (CHO-E) were a generous gift from Dr. Robert Sackstein (Harvard Medical School, Boston, MA). CHO-E cells were maintained in MEM supplemented with 10% FBS and 0.1 mM non-essential amino acids (Life Technologies). Human umbilical vein endothelial cells (HUVECs) were obtained from Lonza, Inc. (Allendale, NJ) and cultured in Medium 199 (Lonza, Inc.) supplemented with 10% FBS, 50 µg/ml endothelial mitogen (Biomedical Technologies, Stoughton, MA), 50 µg/ml heparin (Sigma-Aldrich, St. Louis, MO), 2 mM L-glutamine, and 1x penicillin-streptomycin. One day prior to experiments, HUVECs were plated in sterilized 6.5 mm diameter flexiPERM gaskets (Greiner Bio-one, Monroe, NC) placed at the center of 35 mm tissue culture dishes [35].

### Antibodies

All primary antibodies were monoclonal antibodies (mAbs), unless otherwise noted. Anti-human CD43 (1G10), CD44 (515), CD62E (68-5H11), CD66 (COL-1), PSGL-1 (KPL-1), HECA-452 (anti-cutaneous lymphocyte antigen recognizing sLe^x^, sLe^a^, and related sialofucosylated moieties), sLe^x^ (CSLEX-1), and all isotype controls were obtained from BD Biosciences (San Jose, CA). Anti-human Mac-2BP mAb (Sp-2) [36] was from eBioscience (San Diego, CA), and anti-human Mac-2BP polyclonal antibody (pAb) [37] and anti-PCLP (3D3) antibodies were purchased from Santa Cruz Biotechnology (Santa Cruz, CA). Recombinant mouse E-selectin/human Fc chimera (E-Ig chimera) was from R & D Systems (Minneapolis, MN). Anti-sLe^a^ antibody (KM-231) was from Calbiochem (San Diego, CA). Fluorescein isothiocyanate (FITC)-conjugated and alkaline phosphatase (AP)-conjugated polyclonal secondary antibodies were from Southern Biotech (Birmingham, AL). AlexaFluor 488- and AlexaFluor 568-conjugated secondary antibodies were obtained from Life Technologies.

### Enzyme Treatments and Cell Surface Biotinylation

Breast cancer cells were treated with 0.1 U/ml *Vibrio Cholerae* neuraminidase (Roche Biochemicals, Indianapolis, IN) for 60 min at 37°C to cleave terminal sialic acid residues. Cell surface proteins were removed by treating cells with a general protease, bromelain (Sigma-Aldrich), at 1% for 60 min at 37°C [5], [23]. After enzyme treatment cells were washed and incubated with 0.1% BSA to block non-specific interactions. Cell surface proteins were biotinylated by EZ-link sulfo-NHS-LC-biotin kit (Pierce Biotechnology, Rockford, IL) according to the manufacturer’s protocol.

### Flow Cytometry

All antibody solutions were prepared at 10 µg/ml concentration in blocking buffer (0.1% BSA in Dulbecco’s phosphate buffered saline; DPBS). Cells were washed with blocking buffer and incubated with primary antibody or isotype control, to determine the background fluorescence level, for 30 min at 4°C. Cells were washed and incubated with secondary antibody for 30 min at 4°C [4], [23]. Finally, cells were washed and analyzed using a FACSAria Special Order Research Product flow cytometer/sorter (BD Biosciences).

### RNA Interference

Mac-2BP knockdown of ZR-75-1 cells was performed using MISSION shRNA lentiviral transduction particles (Sigma-Aldrich, St. Louis, MO) prepared from pLKO.1 vector (TRC 1 version). The cell transductions with viral particles containing empty vector or specific sequence were carried out at an optimized concentration of viral particles (20 multiplicity of infection; MOI). From a library of five constructs, a construct (5′- CCGGGTACTTCTACTCCCGAAGGATCTCGAG*ATCCTTCGGGAGTAGAAGTAC*TTTTT-3′; underlined portion is sense and italic portion is antisense sequence; TRCN0000029415) that produced significantly high levels of knockdown of Mac-2BP as determined by flow cytometry was chosen.

### Parallel Plate Flow Chamber Adhesion Assay

The flow adhesion assays were performed using a parallel plate flow chamber (Glycotech, Rockville, MD) placed on a Nikon TE300 inverted microscope equipped with a CCD video camera. Prior to experiments HUVECs were activated to express E-selectin by treatment with 50 U/ml interleukin-1β (IL-1β; Calbiochem) at 37°C for 6 hr [5], [23]. In certain experiments, activated HUVECs were treated with anti-CD62E mAb to block E-selectin function. Breast cancer cells with or without enzyme treatment were perfused over activated or activated and anti-CD62E mAb treated HUVECs. All experiments were performed at a bone marrow microvasculature shear rate of 80 s^−1^ [38], equivalent to 0.8 dynes/cm^2^ in our system, and recorded for 2 min for later analysis. The number of adhering cells included all cells attaching from the free fluid stream [5], [23].

In the other flow adhesion experiments designed for cell detachment analysis, wild type, empty vector transduced or Mac-2BP shRNA transduced ZR-75-1 cells were perfused over a monolayer of CHO-E cells for 4 min at a shear rate of 80 s^−1^. Subsequently, the shear rate was doubled in time steps of 30 s up to the final shear rate of 2560 s^−1^. The numbers of adhering cells corresponding to each shear stress were counted at the end of the 30 s time intervals. The percentage of attached cells was found with respect to attached cells at 80 s^−1^. Cell velocity was calculated by capturing still images from a video over 5 s intervals and measuring cell displacement using Image J software [5].

### Cell Lysis and Immunoprecipitation

Cell lysates were prepared in lysis buffer containing 1% Triton X-100, 0.02% NaN_3_, 150 mM NaCl, 0.5 mM Tris (pH 10.4), 1 mM EDTA and protease inhibitor cocktail (Roche Applied Sciences, Indianapolis, IN). To immunoprecipitate Mac-2BP, cell lysates were incubated with anti-Mac-2BP mAb [39] and protein G agarose beads (Life Technologies) overnight at 4°C under constant rotation. Antigen-antibody-bound protein G beads were subsequently washed with lysis buffer, and the beads were then incubated with Laemmli reducing sample buffer and heated to 100°C for 5 min to release the Mac-2BP. The immunoprecipitates were subsequently subjected to SDS-PAGE and Western blotting [16].

To immunoprecipitate E-selectin reactive proteins, cell lysates were prepared in a modified lysis buffer containing 1 mM CaCl_2_ but no EDTA. To reduce non-specific binding to the human Fc portion of E-Ig chimera, lysate was pre-cleared by incubating with human IgG (h-IgG) isotype control (5 µg/10 million cell lysate) and protein G beads. The pre-cleared cell lysate was then incubated overnight with E-Ig chimera (5 µg/10 million cell lysate) and protein G beads at 4°C with constant rotation. After sufficiently washing antigen-antibody-bound protein G beads, E-Ig chimera reactive antigens were eluted using elution buffer (5 mM EDTA, 50 mM Tris (pH 7.4), and 0.1% Triton-X-100). Eluted samples were subjected to SDS-PAGE and Western blotting.

### SDS-PAGE and Western Blotting

Cell lysates or immunoprecipitates were resolved on 4–15% Tris-HCl precast gels (Bio-Rad Laboratories, Hercules, CA) by reducing SDS-PAGE and subsequently transferred to polyvinylidene difluoride (PVDF; Bio-Rad) membrane [4], [16]. Western blots were probed with appropriate antibodies or isotype controls and AP-conjugated secondary antibodies.

### Mass Spectrometry Analysis of E-selectin Reactive Protein

E-Ig chimera immunoprecipitate isolated from ZR-75-1 cell lysate was resolved by SDS-PAGE. In parallel, E-Ig chimera immunoprecipitate from surface biotinylated cell lysate was Western blotted with streptavidin-AP to serve as a reference for protein migration. The band corresponding to a molecular weight of 72 kDa was excised and sent to Protea Biosciences (Morgantown, WV) for mass spectrometry analysis. The gel fragments were digested with trypsin and were analyzed by liquid chromatography interfaced to matrix assisted laser desorption/ionization-time of flight-mass spectrometry (LC-MALDI-TOF-TOF MS/MS). The mass spectrometry data was subjected to the ProGroup algorithm in Applied Biosystems ProteinPilot 3.0 software, Paragon search engine, and Swissprot database. The protein confidence expressed as Prot Score was more than 99% significant with p<0.01 [40].

### Immunofluorescence Microscopy and Image Deconvolution

Cells were labeled with primary antibody or isotype control as described for flow cytometry. After primary incubation, the cells were washed and incubated with appropriate AlexaFluor 488 (green)-and/or AlexaFluor 568 (red)- conjugated secondary antibody at 4 µg/ml for 30 min at 4°C. After washing, cells were fixed in 4% methanol-free paraformaldehyde in DPBS [41]. The cells were mounted on glass slides in ProLong Gold antifade reagent with DAPI (Life Technologies).

Breast cancer tissue slides were prepared for fluorescence immunohistochemistry as follows. Formalin fixed paraffin embedded (FFPE) breast invasive ductal carcinoma tissue slides (US Biomax, Rockville, MD) [42] were deparaffinized by heating at 60°C for 30 min, and serially incubating with xylene (three times, 10 min each), 95% ethanol, 70% ethanol, and deionized water (once, 5 min each) [43]. For antigen retrieval, the deparaffinized tissue slides were heated at 95°C for 30 min with 10 mM sodium citrate at pH 6.0. The tissue slides were blocked with 1% BSA and 1% FBS in DPBS for 30 min, and incubated for 1 hour with HECA-452 mAb [21] and anti-Mac-2BP pAb [37] at 5 µg/ml and 10 µg/ml, respectively. The slides were washed and incubated with appropriate AlexaFluor conjugated secondary antibodies at 4 µg/ml for 30 min at room temperature. After washing, the tissue slides were mounted in ProLong Gold antifade reagent for microscopy [43].

Tissue and cell slides were imaged using 10× or 40× objectives, respectively, under wide field fluorescence using a Leica DMI 6000 inverted microscope (Leica Microsystems, Wetzlar, Germany) equipped with a motorized high precision specimen stage and an automated optical filter cube wheel with appropriate excitation and emission filters. Images were acquired using Simple PCI software (Hamamatsu Corporation, Sewickley, PA), and were subjected to 2D or 3D blind deconvolution algorithms in AutoQuant X software (Media Cybernetics, Bethesda, MD) to reduce out of focus light. Image projections of processed 3D images were also generated using AutoQuant X [41]. Manders’ overlap coefficient, which indicates quantitative co-localization on the scale of 0 (no overlap) to 1 (100% overlap), was found by using the JACoP plugin in ImageJ 1.40 g software. The thresholds for the calculations were set to automatically retrieved background values [44].

### Statistics

Data are expressed as mean ± SE for at least 3 independent experiments except where indicated. Statistical significance of differences between means was determined by paired Student’s t-test, and probability values of P≤0.05 were considered statistically significant.

## Results

### Adhesion of ZR-75-1 Breast Cancer Cells to HUVECs is Mediated by E-selectin

**Figure 1:**
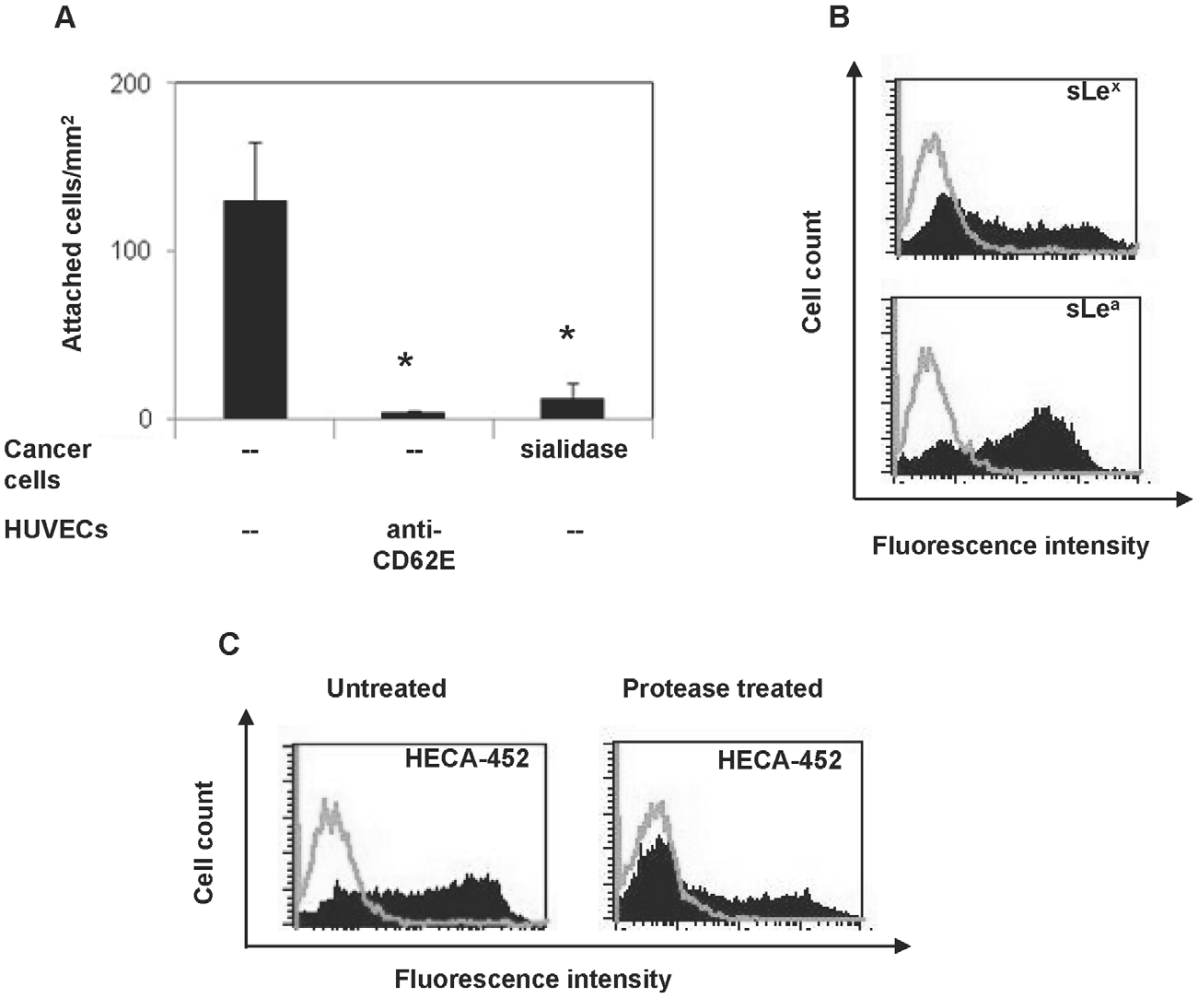
ZR-75-1 cell adhesion to IL-1β activated HUVECs is E-selectin mediated. (**A**) ZR-75-1 cells (10^6^/ml) were perfused over activated (left bar) or anti-CD62E mAb treated activated HUVECs (middle bar), or sialidase (neuraminidase; 1% at 37°C for 30 min) treated ZR-75-1 cells were perfused over activated HUVECs (right bar) for 2 min at a wall shear rate of 80 s^−1^. Data are mean ± SE for n = 3–6 independent experiments. *P<0.05 with respect to untreated cells control. (**B**) ZR-75-1 cells were surface labeled with anti-sLe^x^ (CSLEX-1) or anti-sLe^a^ (KM-231) mAbs and analyzed by flow cytometry. Open curves show isotype and filled curves show specific mAb. (**C**) Untreated or protease treated (1% bromelain at 37°C for 1 hr) ZR-75-1 cells were surface labeled with HECA-452 mAb and analyzed by flow cytometry. Open curves show isotype, and filled curves show specific mAb.

The E-selectin ligand activity of the bone metastatic ZR-75-1 breast carcinoma cell line was tested by perfusing the breast cancer cells over IL-1β activated HUVECs under physiological flow conditions in the parallel plate flow chamber adhesion assay. The breast cancer cells attached to activated HUVECs, but the binding was completely diminished when the HUVECs were treated with E-selectin function blocking mAb (Figure 1A), demonstrating that the adhesion of ZR-75-1 cells to HUVECs is specifically mediated by E-selectin. When sialidase treated ZR-75-1 cells were perfused over activated HUVECs, the adhesion of treated ZR-75-1 cells was significantly reduced as compared to the adhesion of untreated cells (Figure 1A), showing the need for sialylated glycans for optimal adhesion of cancer cells. The data are consistent with previously reported data that terminal sialylation is necessary for E-selectin ligand function [5], [6], [21], [23]. Collectively, these results demonstrate that the adhesion of ZR-75-1 cells to activated endothelium is specifically mediated by the binding of sialylated ligands to endothelial E-selectin.

Expression of sialylated oligosaccharides on breast cancer cells that are indicative of E-selectin ligand activity [5], [21], [23] was tested by flow cytometric analysis. ZR-75-1 cells stained with anti-sLe^x^ (CSLEX-1) and anti-sLe^a^ (KM-231) mAbs showed positive expression for both types of antigens (Figure 1B). Furthermore, ZR-75-1 cells were reactive with HECA-452 mAb (Figure 1C), which broadly recognizes sialofucosylated molecules [21], [22]. Thus, ZR-75-1 cells express classical E-selectin binding sialofucosylated epitopes, including sLe^x^ and sLe^a^.

**Figure 2:**
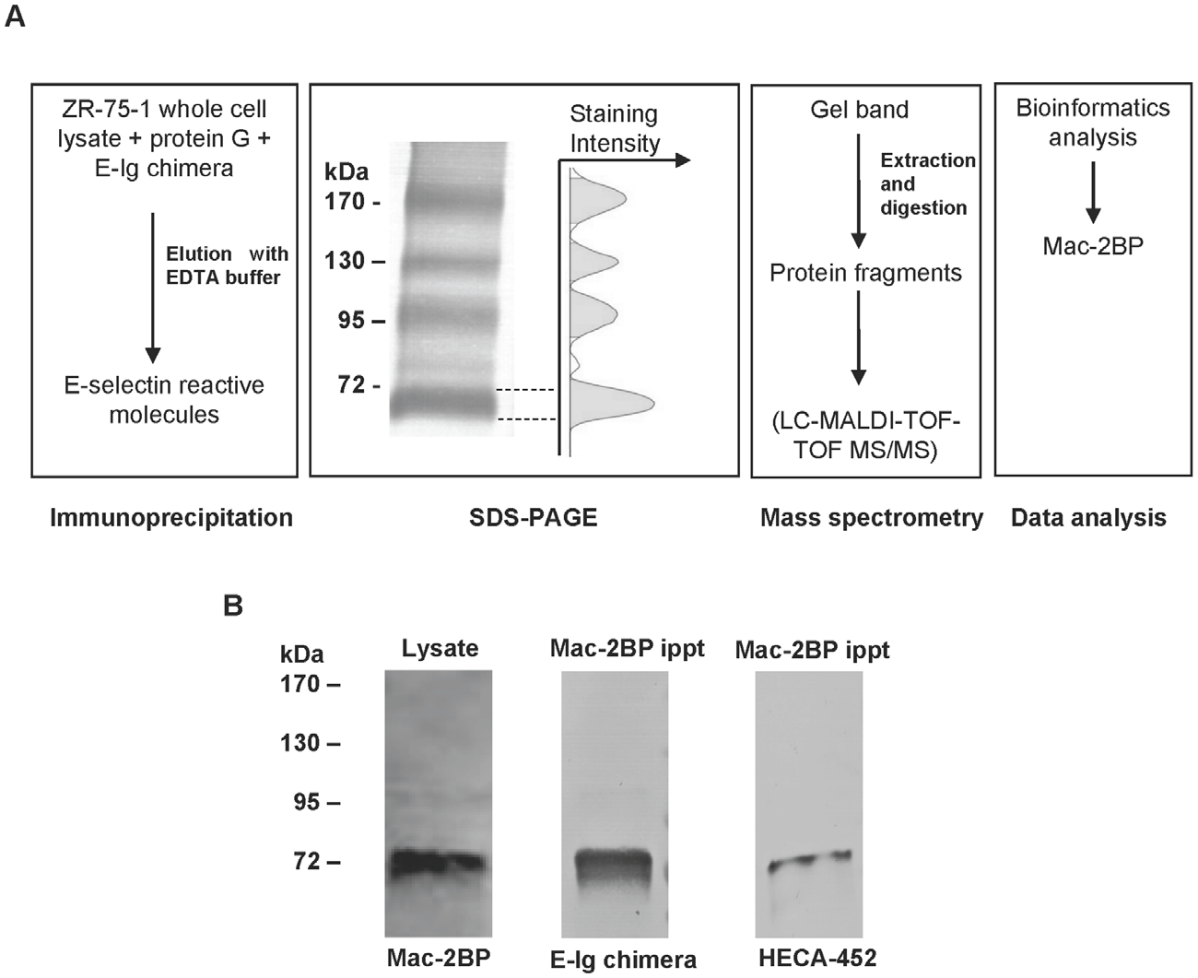
Identification of a novel protein, Mac-2BP, as an E-selectin ligand. (**A**) The steps followed for identification of the E-selectin reactive protein. Biotinylated ZR-75-1 cells (5 × 10^6^) were lysed and immunoprecipitated using E-Ig chimera. Immunoprecipitates were resolved by SDS-PAGE and subsequently blotted with streptavidin-AP. The intensity histogram was obtained by analysis of digitalized image using Image Lab software. (**B**) Lysate from ZR-75-1 cells (2 × 10^6^) was subjected to Western blotting with anti-Mac-2BP pAb. Immunoprecipitates from ZR-75-1 cell lysate (5 × 10^6^ cells) by anti-Mac-2BP mAb were Western blotted with E-Ig chimera or HECA-452 mAb.

### ZR-75-1 Cells do not Express Known E-selectin Ligands

We have previously shown that BT-20 and MDA-MB-468 breast cancer cell lines express ganglioside E-selectin ligands [23]. However, ZR-75-1 gangliosides did not possess detectable E-selectin ligand activity by immuno-overlay and lipid perfusion assays (data not shown), techniques described previously [23]. Consistent with these results, ZR-75-1 cells treated with bromelain, a general protease, showed reduced HECA-452 mAb reactivity compared to untreated cells when tested by flow cytometry (Figure 1C), indicating that proteins, rather than lipids, preferentially express appropriate carbohydrate modifications required for E-selectin ligand activity. The efficacy of bromelain treatment in these experiments was confirmed by loss of expression of E-cadherin, which was nearly reduced to isotype control levels after treatment (data not shown).

The absence of candidate glycolipid ligands implied that glycoproteins are the major E-selectin ligands expressed by ZR-75-1 cells. However, known glycoprotein E-selectin ligands PSGL-1, CD43, CD44, CD66, and PCLP [2], [4], [16], [17], [18], [19], [20] were not detected on ZR-75-1 cells by flow cytometric analysis (data not shown). These results strongly suggested that ZR-75-1 cells may express previously unidentified glycoprotein ligands.

**Figure 3:**
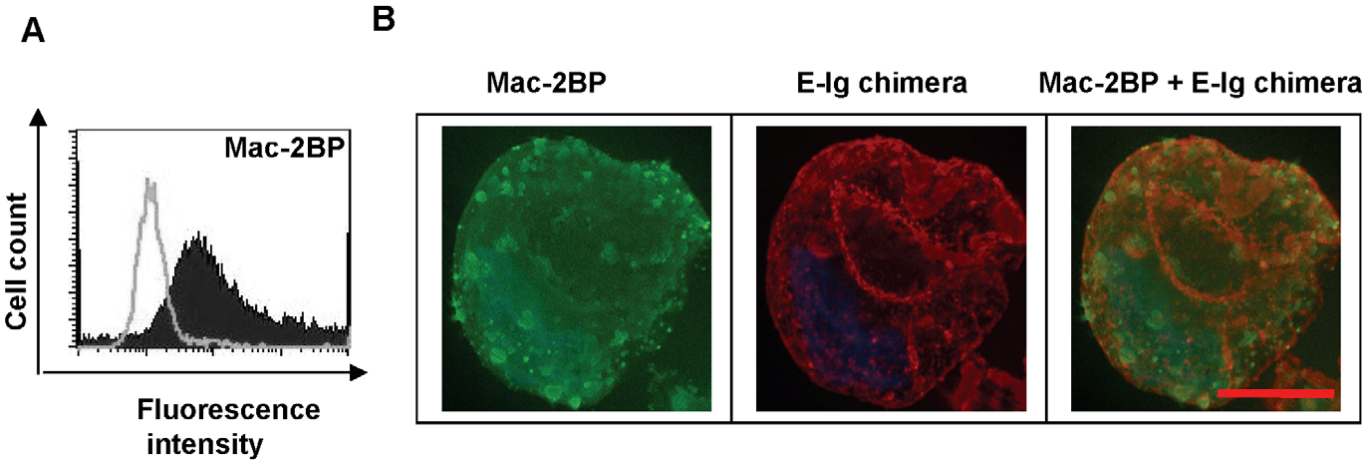
Immunostaining of ZR-75-1 cells shows co-localization of signals for Mac-2BP and E-selectin ligand activity. (**A**) ZR-75-1 cells were surface labeled with anti-Mac-2BP pAb and analyzed by flow cytometry. Open curve shows isotype, and filled curve shows specific antibody. (**B**) ZR-75-1 cells were dually surface labeled with anti-Mac-2BP pAb (green) and E-Ig chimera (red). Images of slices, 0.5 µm apart, were obtained in epifluorescence microscopy, and projected to obtain a composite image. The composite image was deconvoluted using AutoQuant X software. Co-localization of two molecules is shown in the overlapped image (orange). Scale bar indicates 10 µm.

### Identification of a Novel E-selectin Ligand

To screen for glycoprotein E-selectin ligands, E-Ig chimera immunoprecipitates obtained from lysates of surface biotinylated cells were Western blotted with streptavidin-AP. Several bands were revealed (Figure 2A), confirming that ZR-75-1 cells express protein E-selectin ligands. The band corresponding to a molecular weight of 72 kDa showed the highest E-selectin activity as found by image intensity analysis of the Western blot (Figure 2A). To identify this protein, the portion of the gel corresponding to the 72 kDa immunoprecipitated protein from ZR-75-1 cell lysate was excised and submitted for mass spectrometry analysis. Multiple peptides matched to Mac-2BP, generating a ProtScore equivalent to 99.99% confidence for the protein identification. Previously, a similar form of Mac-2BP (∼75 kDa) was found to be expressed by other cancer cell lines and tissues [39], [45], [46] and was shown to be a cell surface form of the protein that is distinct from the secreted form of Mac-2BP (∼90 kDa) [39].

**Figure 4:**
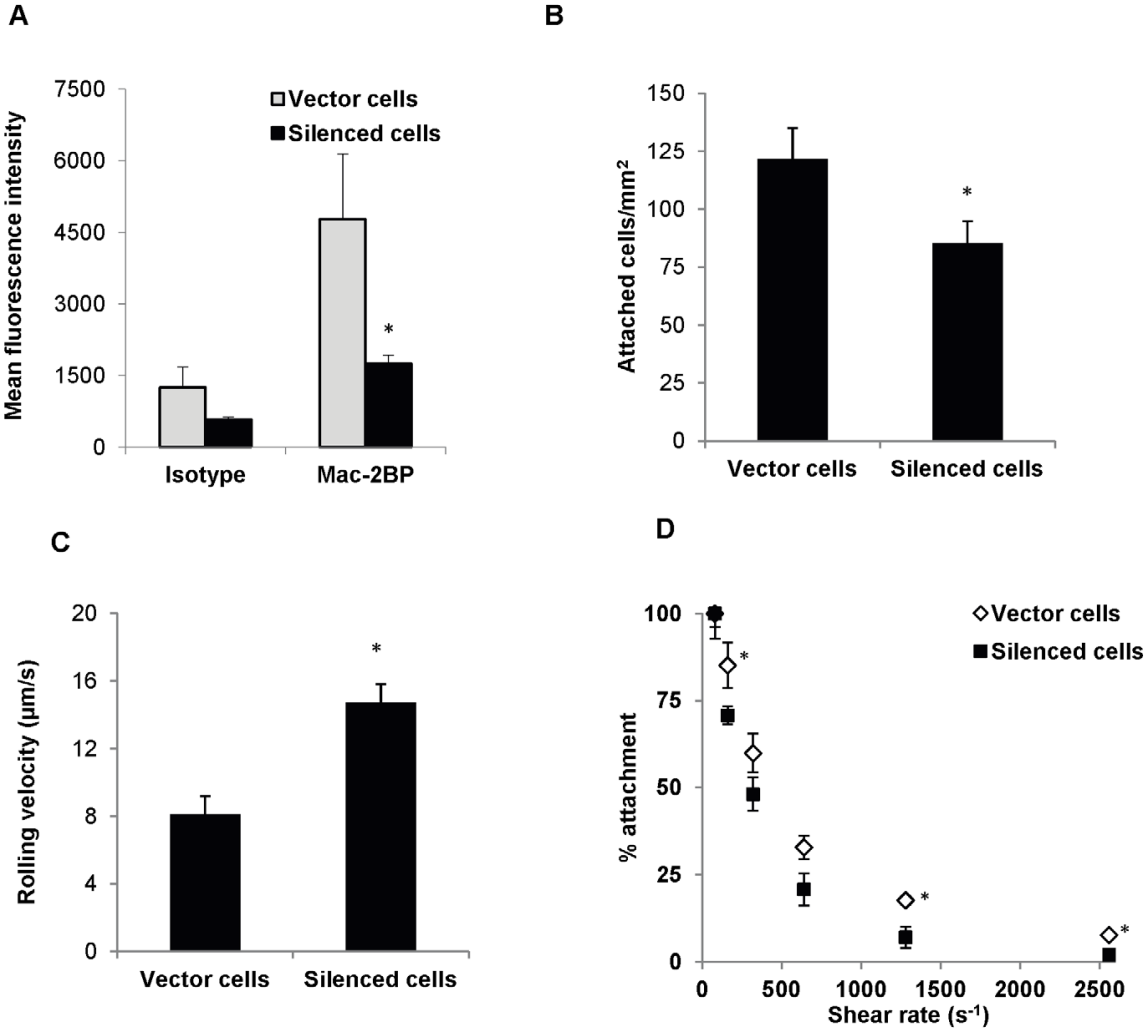
Mac-2BP silencing of ZR-75-1 cells reduces their adhesion to E-selectin. (**A**) Vector or Mac-2BP silenced cells were surface labeled with anti-Mac-2BP pAb and analyzed by flow cytometry. The data are represented as mean fluorescence intensity ± SE for n = 5 independent experiments. *P<0.05 with respect to vector. (**B**) Vector or Mac-2BP silenced cells (10^6^/ml) were perfused over CHO-E cells for 4 min at a wall shear rate of 80 s^−1^ and the number of adhering cancer cells were counted. Data are mean ± SE for n = 5 independent experiments. *P<0.05 with respect to vector. (**C**) The rolling velocity of vector and silenced cells over CHO-E cells was determined at a wall shear rate of 80 s^−1^. Data are mean ± SE for n = 10 cells. *P<0.05 with respect to vector. (**D**) Vector or silenced cells (10^6^/ml) were perfused over CHO-E cells for 4 min at a wall shear rate of 80 s^−1^ and then the shear rate was doubled in 30 s time intervals. Data are mean ± SE for n = 5 independent experiments. *P<0.05 with respect to vector.

### Mac-2BP Expressed by ZR-75-1 Cells is an E-selectin Ligand

To confirm breast cancer cell expression of Mac-2BP and its E-selectin ligand activity, a series of Western blots were performed. ZR-75-1 cell lysate probed with Mac-2BP antibody revealed a band corresponding to 72 kDa (Figure 2B), verifying the expression of Mac-2BP at the same molecular weight of the major E-Ig immunoprecipitated protein. Furthermore, staining of immunoprecipitated Mac-2BP by E-Ig chimera or HECA-452 mAb (Figure 2B) showed that Mac-2BP expressed by ZR-75-1 cells is a sialofucosylated E-selectin ligand, confirming the results described earlier (Figures 1 and 2A).

Since functional E-selectin ligands must be natively exposed on the cell surface, the expression of Mac-2BP on ZR-75-1 cells was tested by flow cytometry. As shown in Figure 3A, ZR-75-1 cells were positive for Mac-2BP compared to the isotype control. To demonstrate that the cell surface Mac-2BP possessed E-selectin binding epitopes, fluorescence microscopy of ZR-75-1 cells with anti-Mac-2BP pAb and E-Ig chimera was performed. The results revealed positive cell surface expression of Mac-2BP as well as E-Ig chimera reactivity compared to the respective isotype controls (Figures 3 and S1). Moreover, the overlapped image (Figure 3B) shows distinct co-localization of Mac-2BP and E-selectin ligand activity. The quantitative analysis by Manders’ coefficient showed that more than 85% of Mac-2BP (green) co-localizes with E-selectin ligands (red), implying the majority percentage of Mac-2BP may possess E-selectin ligand activity. The analysis also indicated that more than 55% of E-selectin ligands overlap with Mac-2BP, implying that the majority of Mac-2BP possesses E-selectin ligand activity, as well as suggesting the presence of other E-selectin ligands, in support of the earlier results (Figure 2A). Together, these data provide further evidence that Mac-2BP expressed by ZR-75-1 cells is a cell surface glycoprotein that possesses E-selectin ligand activity.

**Table 1:** Diverse breast cancer cell lines possess E-selectin ligand activity.

Cell line	Tumor type and stage [50]	Specimen site [50]	sLe^x^	sLe^a^	E-selectin ligand activity
**ZR-75-1**	IDC (IV)	Ascites	++	++	++
**BT-20**	IDC (NR)	Primary tumor	++	++	++
**MDA-MB-468**	AC (IV)	Pleural effusion	+	−	++
**T-47D**	IDC (IV)	Pleural effusion	+	−	+
**MDA-MB-231**	IDC (IV)	Pleural effusion	+	+	+
**MCF-7**	IDC (IV)	Pleural effusion	−	+	++
**Hs-578t**	IDC (NR)	Primary tumor	+	−	+

Breast cancer cells were surface labeled with anti-sLe^x^ mAb (CSLEX-1), anti-sLe^a^ mAb (KM-231), or E-Ig chimera and analyzed by flow cytometry. The positive mean fluorescence intensities, compared to respective isotype control, are categorized as high (++) or low (+). The negative/very low intensities with respect to respective isotype control are indicated by (−). IDC, invasive ductal carcinoma; AC, adenocarcinoma; NR, not reported.

**Figure 5:**
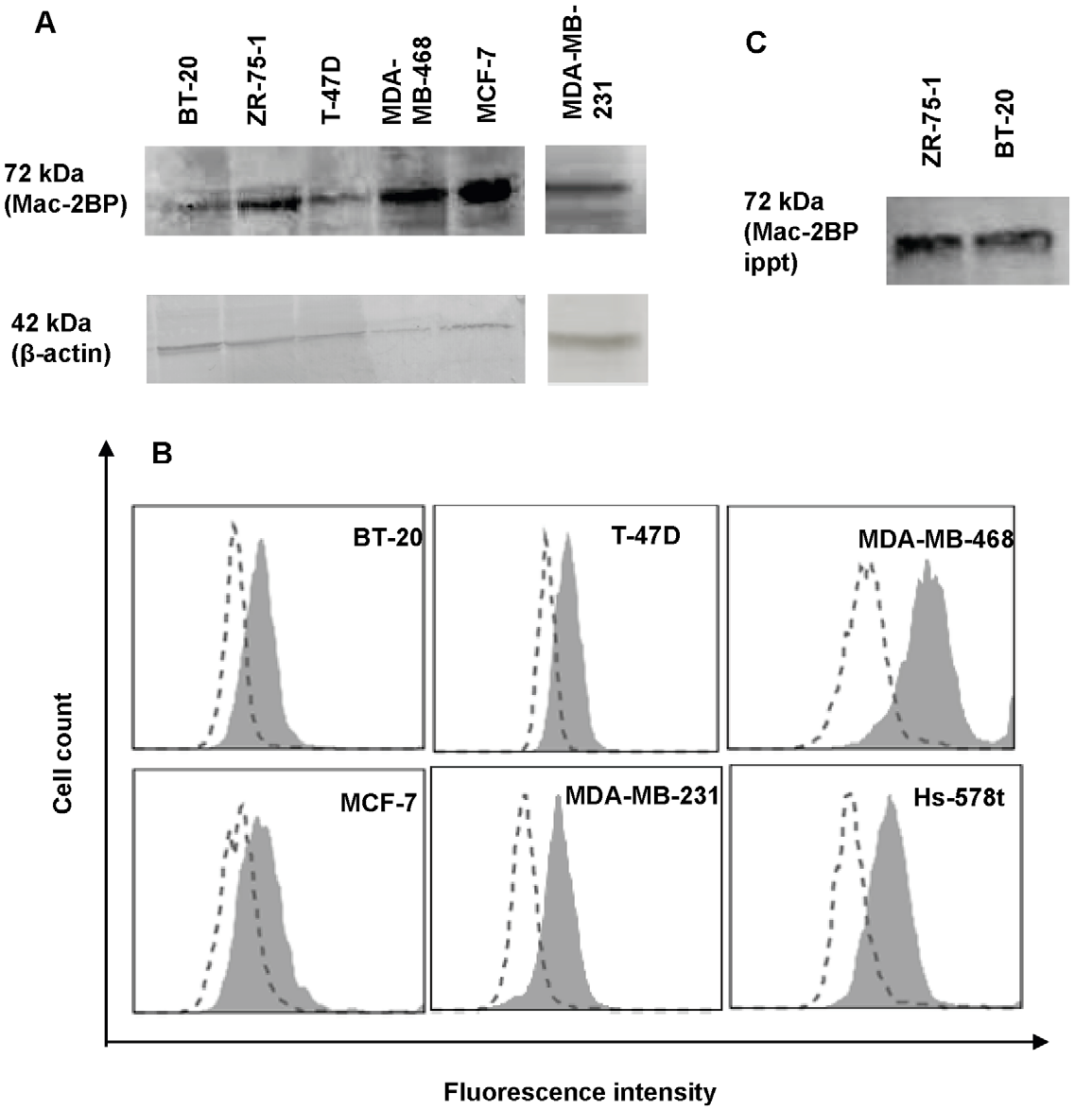
Several breast cancer cell lines express Mac-2BP. (**A**) Lysates of 2×10^6^ cells obtained from a variety of breast cancer cell lines were subjected to Western blot analysis with anti-Mac-2BP pAb. β-actin staining was used as the loading control. (**B**) Breast cancer cells were surface labeled with anti-Mac-2BP pAb and analyzed by flow cytometry. Open curve shows isotype, and filled curve shows specific antibody. (**C**) Immunoprecipitates from ZR-75-1 or BT-20 cell lysate (5 × 10^6^ cells) by anti-Mac-2BP mAb were subjected to Western blotting with E-Ig chimera.

**Figure 6:**
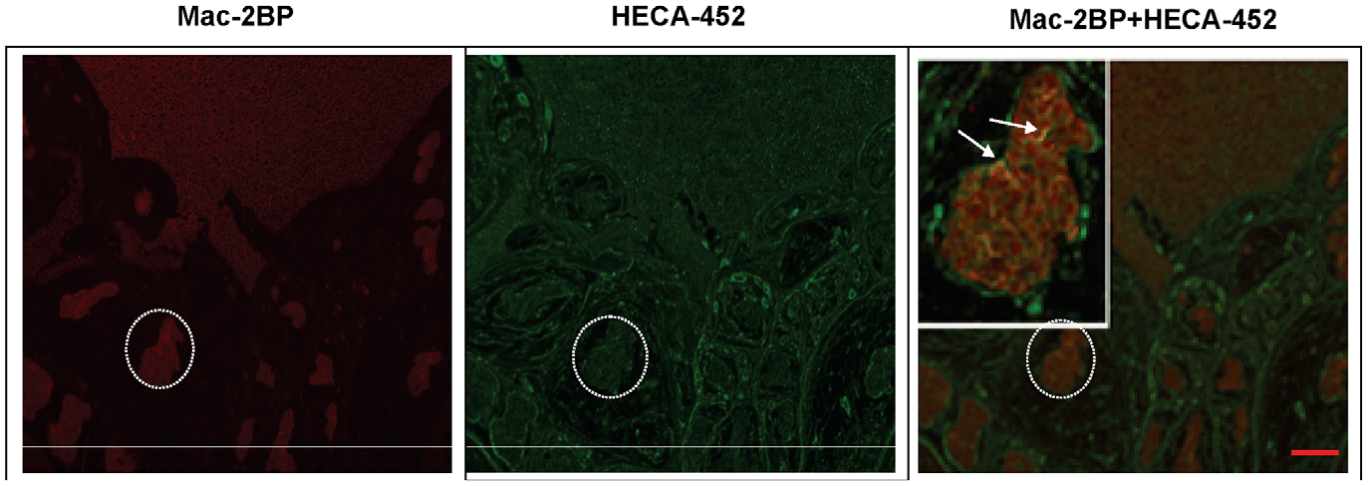
Immunofluorescence analysis shows co-localization of Mac-2BP and E-selectin ligand activity on breast cancer tissue. Deparaffinized breast invasive ductal carcinoma tissue was labeled with anti-Mac-2BP pAb (red) and HECA-452 mAb (green). Co-localization of two signals is shown in the overlapped image (orange). Scale bar indicates 100 µm.

### Mac-2BP Regulates E-selectin Mediated Rolling and Adhesion of ZR-75-1

To investigate the native E-selectin ligand activity of Mac-2BP, ZR-75-1 cells were transduced with shRNA for Mac-2BP, or empty vector as a negative control, and tested in parallel plate flow chamber adhesion assays. Mac-2BP silenced cells, compared to vector cells, expressed significantly reduced levels of Mac-2BP (Figures 4A and S2), showing an efficient knockdown. In experiments performed to test adhesion specificity, transduced ZR-75-1 cells perfused at shear rate of 80 s^−1^ adhered to CHO-E cells, but the adhesion was completely abrogated when CHO-E cells were treated with E-selectin function blocking mAb (data not shown), indicating that the adhesion was specifically mediated by E-selectin. Interestingly, the adhesion of Mac-2BP silenced cells (85±9 cells/mm^2^) to CHO-E cells at a shear rate of 80 s^−1^ was significantly less than that of vector cells (121±13 cells/mm^2^; Figure 4B), demonstrating that Mac-2BP is a crucial E-selectin ligand under physiological flow conditions. Furthermore, the rolling velocity of silenced cells was significantly higher than that of vector cells (Figure 4C), demonstrating the role of Mac-2BP-E-selectin ligation in controlling cell rolling velocity, a classical function of E-selectin ligands [5], [47]. Similar levels of adhesion and rolling velocities were found for wild type ZR-75-1 and vector cells (data not shown), indicating that the cell transduction did not non-specifically alter cell adhesion function. For detachment analysis, which was performed by sequentially increasing the shear rate from 80 s^−1^, the adhesion data of vector and silenced cells were normalized with number of attached cells of respective types at 80 s^−1^ (Figure 4D). A consistently lower percentage attachment of silenced cells, relative to that of vector cells, at all shear rates studied was observed (Figure 4D). These data collectively demonstrate Mac-2BP as an E-selectin ligand necessary for regulating cell adhesion under hemodynamic flow conditions.

The E-selectin ligand activity of Mac-2BP was also found in experiments performed using IL-1β activated HUVECs as E-selectin expressing cells. A significantly lower number of Mac-2BP silenced cells (34±4 cells/mm^2^) compared to vector cells (58±9 cells/mm^2^), attached to the HUVECs, consistent with CHO-E data (Figure 4B). Yet no significant difference was found between the rolling velocities of vector and silenced cells (9.0±1.0 versus 9.1±1.3 µm/s, respectively). These results may indicate alternative molecular pathways for regulating rolling of ZR-75-1 cells to activated endothelium, and these data are consistent with other reports suggesting alternative cell adhesion pathways for cancer cell adhesion to activated endothelium [5], [48], [49].

**Figure 7:**
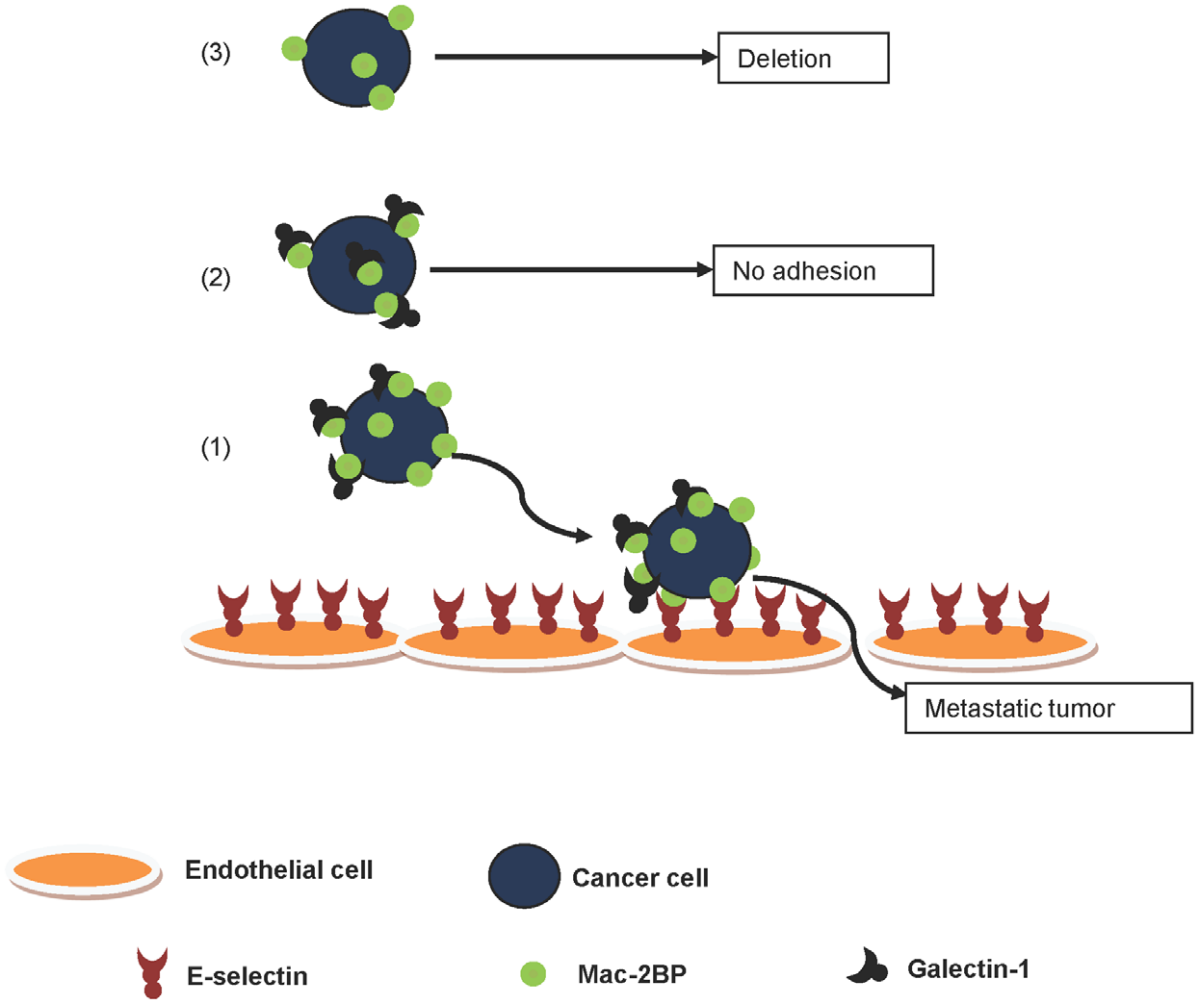
Hypothesized model for the role of Mac-2BP in metastasis. Three possible scenarios for Mac-2BP expressing breast cancer cells. (1) Breast cancer cells expressing high levels of Mac-2BP bind to Gal-1 yet possess enough free epitopes for E-selectin binding. Thus, these cells are more likely to form metastatic lesions than (2) cells expressing low levels of Mac-2BP, which may not bind to endothelium due to blockade of E-selectin ligand function by Gal-1. (3) Absence of Gal-1 may lead to detection and deletion of cancer cells by immune cells.

### Mac-2BP is Expressed by Several Other Breast Cancer Cell Lines that also Possess E-selectin Ligand Activity

Although identification of a ubiquitous E-selectin ligand expressed by multiple breast cancer cell lines is lacking, many breast cancer cell lines have been previously shown to possess E-selectin ligand activity [7], [8], [23], [24], [25], [26]. Yet the types of assays and assay conditions used in these studies vary greatly [7], [8], [23], [24], [25], [26]. Therefore, flow cytometry using E-Ig chimera and carbohydrate antibodies was performed to test for E-selectin ligand activity and to compare carbohydrate expression profiles of several widely studied breast cancer cell lines: ZR-75-1, BT-20, MDA-MB-468, T-47D, MDA-MB-231, MCF-7, and Hs-578t. The results showed that all of these cell lines possess detectable E-selectin ligand activities and express carbohydrates indicated in E-selectin ligand activity (Table 1). Further, Western blotting of cell lysates or flow cytometry analysis of cells revealed that all of these cell lines express Mac-2BP (Figures 5A and B). Since BT-20 cells possess high E-selectin ligand activity (Table 1 and [23]) and are derived directly from a primary invasive breast cancer tumor [50], they may be used as a model cell line to validate the E-selectin ligand activity of Mac-2BP expressed in the primary site. Mac-2BP immunoprecipitated from BT-20 cells stained with E-Ig chimera revealed Mac-2BP as an E-selectin ligand, similar to ZR-75-1 cells (Figure 5C).

### Expression of Mac2bp Correlates with E-selectin Ligand Activity in Invasive Breast Cancer Tissue

To investigate the presence of Mac-2BP as an E-selectin ligand in breast cancer tissue, fluorescence immunostaining of breast invasive ductal carcinoma tissue was performed. Parallel sections from breast cancer tissue were dual stained to reveal expression of Mac-2BP and HECA-452 antigens. Tissue staining with specific antibodies was positive compared to respective isotype controls, illustrating that Mac-2BP and HECA-452 antigens are expressed in breast cancer tissue (Figures 6 and S3). A composite image revealed a visibly distinct co-localization of signals (arrow heads in Figure 6), quantification of which by Manders’ coefficient showed that more than 70% of Mac-2BP co-localizes with HECA-452 antigens and more than 40% of sialofucosylated antigens relate with Mac-2BP. Collectively, these results suggest that breast cancer tissue expression of Mac-2BP is related to high affinity E-selectin ligand activity.

## Discussion

In distant metastasis, circulating tumor cells attach to the vasculature of a remote tissue or organ by engaging with endothelial cell adhesion molecules. Although breast cancer cell adhesion to endothelial cells has been shown to be mediated by E-selectin [7], [8], [23], [24], [25], [26], a limited number of reports have characterized breast cancer cell E-selectin ligands. In stark contrast, several E-selectin ligands have been identified on leukemic, colon, and prostate cancer cells [2], [4], [5], [16], [18], [19]. In the present report we bridged this knowledge gap by investigating the E-selectin mediated adhesion and E-selectin ligands of ZR-75-1 breast cancer cells. We have identified and characterized a new glycoprotein ligand, Mac-2BP, which to the best of our knowledge has not been shown previously to possess E-selectin ligand activity. Mac-2BP is upregulated in advanced stage cancer tumors, and its expression positively correlates to development of distant metastasis [31], [51]. Our study thus ascribes a functional role for Mac-2BP in cell adhesion by identifying it as an E-selectin ligand, and potentially explains one of its roles in cancer metastasis.

ZR-75-1 breast cancer cells specifically adhered to endothelial E-selectin under physiological flow conditions (Figure 1A), consistent with other breast cancer cell lines [8], [23]. The E-selectin ligand activity of these cells was associated with sialofucosylated carbohydrates including sLe^x^ and sLe^a^ (Figures 1B and C), which have been reported to provide E-selectin ligand activity to core protein or lipid molecules [16], [21], [52]. Notably, mainly glycoproteins rather than glycolipids were sialofucosylated in ZR-75-1 cells (Figure 1C). Hence, glycoproteins possessed appropriate carbohydrate modifications for E-selectin ligand activity. It is believed that cell surface proteins are preferred E-selectin ligands for the initiation of endothelial adhesion under hematogenous flow conditions. They can extend farther than lipids from the cell surface and therefore can easily make initial contact with endothelial E-selectin. In fact, many human cell lines and native cells have been shown to employ glycoproteins ligands for E-selectin mediated adhesion *in vitro* and *in vivo* [2], [4], [15], [16], [17], [18], [19], [20].

ZR-75-1 cells express Mac-2BP as a heretofore unrecognized E-selectin ligand, but known E-selectin ligands were undetected. Furthermore, several other potentially novel E-selectin ligands were found (Figure 2A). Identification and characterization of these proteins are in progress in our laboratory. The putative ability of Mac-2BP expressed by ZR-75-1 cells to mediate E-selectin binding was investigated by using HECA-452 mAb, which detects sialofucosylated epitopes that are purported to confer high efficiency E-selectin ligand activity. Several investigators have used HECA-452 mAb for analyzing E-selectin ligands. For example, a glycoform of CD44 known as HCELL (hematopoietic cell E−/L-selectin ligand), a major E-selectin ligand expressed by human colon cancer and hematopoietic stem cells, was found to be reactive to HECA-452 mAb by Western blot [6], [16]. A similar approach in our study revealed that Mac-2BP is a high efficiency E-selectin ligand, whose ligand activity is primarily associated with sialofucosylated epitopes detectable by HECA-452 mAb (Figure 2B). However, we do not discount the possible existence of Mac-2BP glycoforms lacking HECA-452 mAb reactivity, especially because some fraction of Mac-2BP expressed in tumor tissue was not reactive to HECA-452 mAb (Figure 6). In agreement with this notion, breast cancer cell lines express diverse carbohydrate profiles yet are positive for E-selectin ligand activity (Table 1 and [23], [24]).

Since Mac-2BP possessed putative structural features necessary for E-selectin ligand activity, the intricate functional details of Mac-2BP expressed on intact cells were unraveled in parallel plate flow chamber adhesion experiments. Selectins and their ligands mediate various steps of adhesion of circulating cells to endothelium [2], [3], [4], [5], [52]. At bone marrow vascular flow conditions, Mac-2BP expressed by ZR-75-1 cells exhibited E-selectin ligand activity necessary for cell capture and controlling cell rolling velocity (Figure 4C). Furthermore, the sequential increase in cell detachment as shear rate was increased suggested that Mac-2BP regulates firm attachment of cells (Figure 4D). In totality, these data clearly demonstrate that Mac-2BP is a potent E-selectin ligand that regulates various stages of the adhesion cascade.

In addition to ZR-75-1 cells, several other breast cancer cell lines possessing E-selectin ligand activities (Table 1) expressed Mac-2BP (Figure 5). The majority of these cell lines metastasize to mouse bone marrow [34], which is known to express E-selectin [38], [53]. In humans, breast cancer frequently spreads to bone marrow [54], and human bone marrow endothelium constitutively expresses E-selectin [55]. These findings lead to the notion that the Mac-2BP-E-selectin cell adhesion pathway is crucial in breast cancer metastasis. In support of this notion, staining of breast carcinoma tissue indicated expression of E-selectin reactive Mac-2BP in invasive breast cancer tumors (Figure 6). Furthermore, previous studies have reported that cancer patients with Mac-2BP over-expressing tumors are more likely to develop distant metastasis, have shorter disease free survival, and have adverse prognosis compared to patients with low Mac-2BP expressing tumors [31], [51]. In association with literature reports, our data imply that Mac-2BP expressing cells in breast tumors may invade distant tissues via Mac-2BP-E-selectin mediated adhesion and suggest E-selectin reactive Mac-2BP as a potential prognostic marker or therapeutic target for prevention of breast cancer metastasis. However, to make such determinations, it would be important to evaluate Mac-2BP-E-selectin mediated adhesion in comparison with other adhesion molecules that may play a role in mediating cancer cell adhesion to activated endothelium [5], [48], [49].

Because Mac-2BP has also been identified previously as a Gal-1 ligand [32], we have hypothesized a unique model for the regulation of breast cancer cell adhesion to endothelium (Figure 7). First, we must revisit leukocyte adhesion to endothelial cells. Leukocytes express specific E-selectin ligands that initiate adhesion to endothelial E-selectin [2], [11], and certain leukocyte subsets express Mac-2BP [56]. It has been reported that soluble Gal-1 inhibits adhesion of leukocytes to activated endothelium under hematogenous flow conditions, although the exact interference mechanisms are unclear [57]. These findings, together with our data that Mac-2BP is an E-selectin ligand, imply that E-selectin-Mac-2BP interactions potentially compete with Gal-1-Mac-2BP binding (Figure 7). In contrast to the inhibitory effect in cell recruitment by blocking E-selectin ligand activity (i.e., prevention of metastasis), Gal-1 is known to help tumor cells to escape from immune action [58]. Therefore, it appears that expression of Mac-2BP by cancer cells is critically regulated, and an optimal expression level of Mac-2BP is required for evading the immune response and to colonize distant tissue. Thus, our results may serve to explain the poor prognosis of breast cancer patients with Mac-2BP over-expressing tumors. Although the present study and the postulated model propose mechanistic links to understand breast cancer metastasis mediated by Mac-2BP, further studies are warranted to confirm and add comprehensive details, including roles for other E-selectin ligands.

In conclusion, our study provides new insights into the molecular mechanisms underlying cell adhesion in breast cancer metastasis. The data show that the adhesion of ZR-75-1 breast cancer cells to endothelial cells is mediated by endothelial E-selectin and Mac-2BP, a novel high efficiency E-selectin ligand. We believe that these interactions lead to other pathways necessary for organotropism of metastasis. Altogether our study, combined with previously reported data, demonstrates that Mac-2BP is an important molecule in breast cancer metastasis, and it is anticipated that further investigation will reveal its prognostic and therapeutic potential.

## Supporting Information

Figure S1 **Immunostaining of ZR-75-1 cells with isotype controls of Mac-2BP and E-Ig chimera is negative compared to that of respective mAbs (shown in manuscript** **Figure 3B** **).** ZR-75-1 cells were dually surface labeled with isotype controls corresponding to anti-Mac-2BP pAb (rabbit IgG) and E-Ig chimera (human IgG). Images of slices, 0.5 µm apart, were obtained in epifluorescence microscopy, and projected to obtain a composite image. The composite image was deconvoluted using AutoQuant X software. Co-localization of two molecules is shown in the overlapped image (rabbit IgG + human IgG). Scale bar indicates 10 µm.
(TIF)

Figure S2 **Histogram overlay of vector and Mac-2BP silenced cells stained with anti-Mac-2BP pAb.** Vector or Mac-2BP silenced cells were surface labeled with anti-Mac-2BP pAb or isotype control and analyzed by flow cytometry. Filled curve shows vector cells, open curve shows Mac-2BP silenced cells labeled with anti-Mac-2BP pAb, and dashed curve shows Mac-2BP silenced cells labeled with isotype control.
(TIF)

Figure S3 **Immunofluorescence staining of breast cancer tissue with isotype controls of Mac-2BP and HECA-452 is negative compared to that of respective mAbs (shown in manuscript** **Figure 6** **).** Deparaffinized breast invasive ductal carcinoma tissue was labeled with isotype controls of anti-Mac-2BP pAb (rabbit IgG) and HECA-452 mAb (rat IgM). Co-localization of two signals is shown in the overlapped image (rabbit IgG + rat IgM). Scale bar indicates 100 µm.
(TIF)
