# Supplementary figures and images for: Mac-2 Binding Protein Is a Novel E-Selectin Ligand Expressed by Breast Cancer Cells
Source: PLoS One. 2012 Sep 6;7(9):e44529. doi: 10.1371/journal.pone.0044529 (PMC3435295; doi:10.1371/journal.pone.0044529)

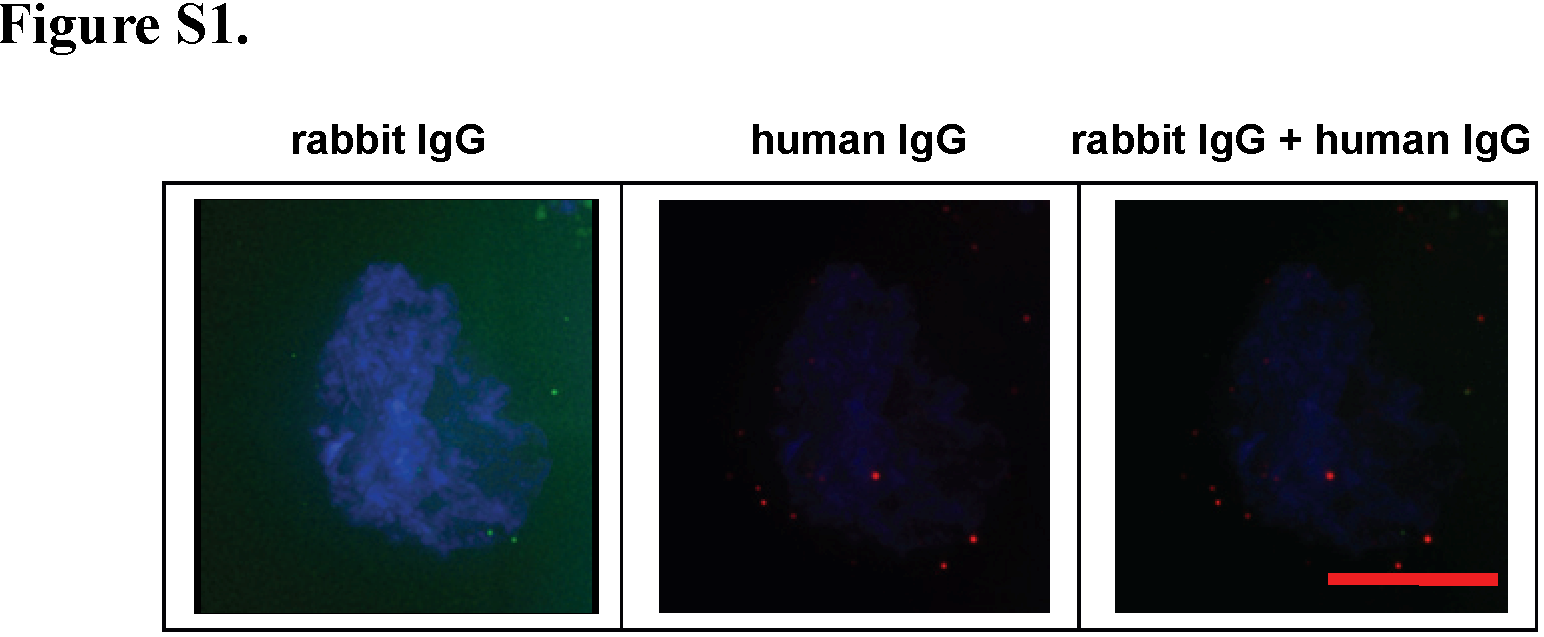

Supplement: Figure S1 — Immunostaining of ZR-75-1 cells with isotype controls of Mac-2BP and E-Ig chimera is negative compared to that of respective mAbs (shown in manuscript Figure 3B ). ZR-75-1 cells were dually surface labeled with isotype controls corresponding to anti-Mac-2BP pAb (rabbit IgG) and E-Ig chimera (human IgG). Images of slices, 0.5 µm apart, were obtained in epifluorescence microscopy, and projected to obtain a composite image. The composite image was deconvoluted using AutoQuant X software. Co-localization of two molecules is shown in the overlapped image (rabbit IgG + human IgG). Scale bar indicates 10 µm. (TIF) [file pone.0044529.s001.tif]

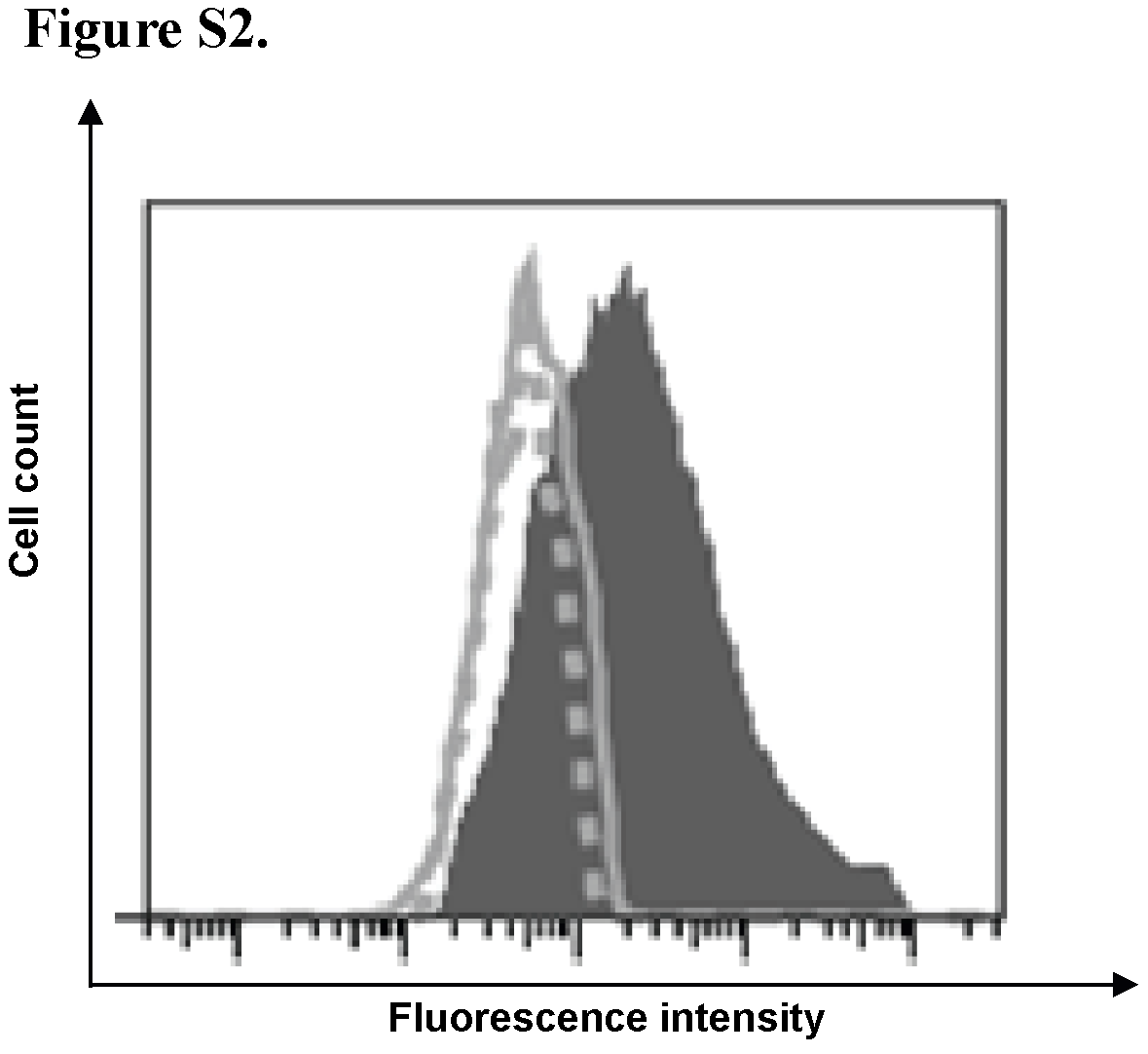

Supplement: Figure S2 — Histogram overlay of vector and Mac-2BP silenced cells stained with anti-Mac-2BP pAb. Vector or Mac-2BP silenced cells were surface labeled with anti-Mac-2BP pAb or isotype control and analyzed by flow cytometry. Filled curve shows vector cells, open curve shows Mac-2BP silenced cells labeled with anti-Mac-2BP pAb, and dashed curve shows Mac-2BP silenced cells labeled with isotype control. (TIF) [file pone.0044529.s002.tif]

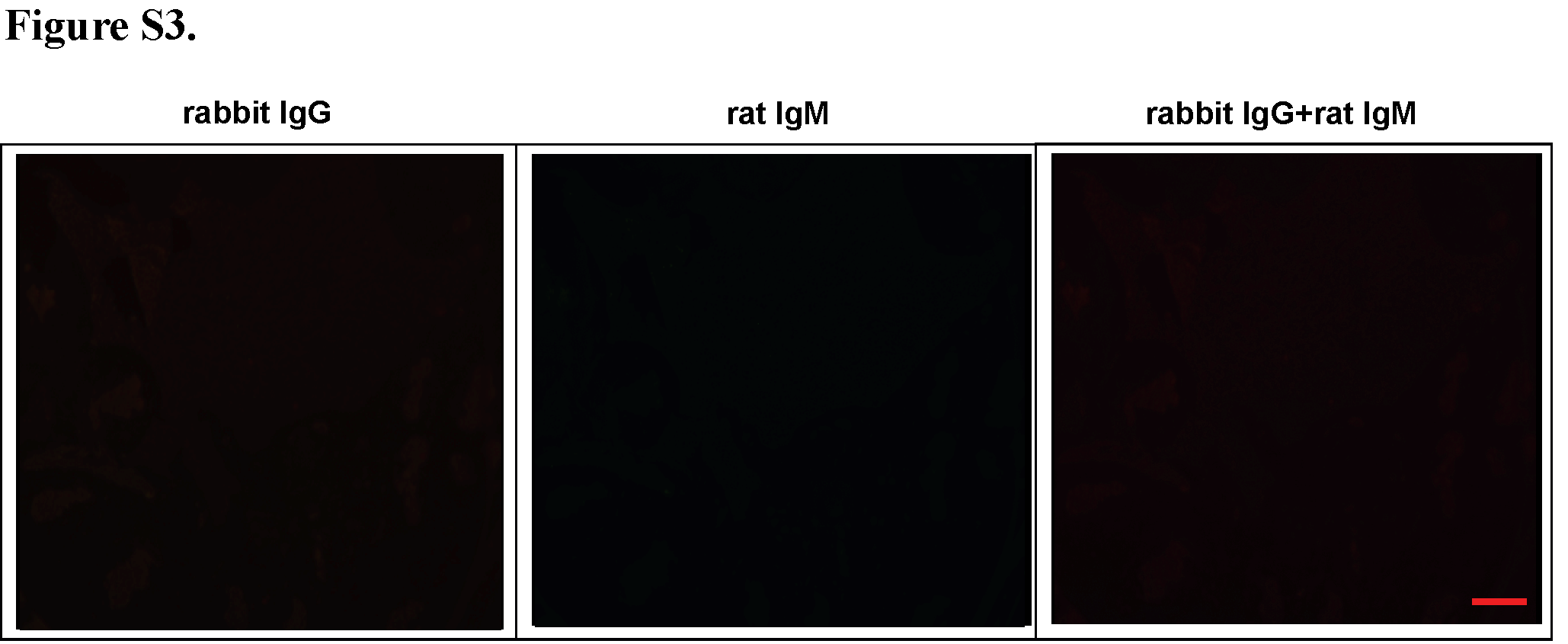

Supplement: Figure S3 — Immunofluorescence staining of breast cancer tissue with isotype controls of Mac-2BP and HECA-452 is negative compared to that of respective mAbs (shown in manuscript Figure 6 ). Deparaffinized breast invasive ductal carcinoma tissue was labeled with isotype controls of anti-Mac-2BP pAb (rabbit IgG) and HECA-452 mAb (rat IgM). Co-localization of two signals is shown in the overlapped image (rabbit IgG + rat IgM). Scale bar indicates 100 µm. (TIF) [file pone.0044529.s003.tif]
